# The Growing Need for Service Provision for People Living with HIV in Bangladesh

**DOI:** 10.3329/jhpn.v28i3.5545

**Published:** 2010-06

**Authors:** Mark Pietroni, Tasnim Azim

**Affiliations:** ^1^ Dhaka Hospital; ^2^ HIV/AIDS Programme ICDDR,B, GPO Box 128, Dhaka 1000, Bangladesh

Despite the fact that the first case of HIV in Bangladesh was detected in 1989, there has been a strong and coordinated approach to preventing HIV and AIDS in the country since 1985. Guided by the National AIDS Committee and its technical subcommittees and led by the National AIDS/STD Programme within the Directorate General of Health Services, Bangladesh has seen the roll out of a National Strategic Plan (NSP) and comprehensive guidelines relating to the prevention and management of HIV and AIDS (1). The Government of Bangladesh deserves to be congratulated on its early and comprehensive response to the issue of HIV and AIDS.

Twenty-one years after the first case was detected, Bangladesh remains a low-prevalence country with an estimated prevalence of <1% in the most at-risk population groups. However, complacency at this point could prove disastrous. Although the numbers remain low, data show a steady rise in the number of new HIV-positive cases, new AIDS cases, and deaths due to AIDS (Table and Fig.). Further, although the overall prevalence among the most at-risk population groups is still <1%, it is 7% among injecting drug-users in Dhaka and rates as high as 10.5% have been reported in a localized neighbourhood in Dhaka (1). More recently, an emerging HIV epidemic has been detected among female sex workers from a town bordering West Bengal, India (2). There is clearly a likelihood of ‘spill over’ or ‘seeding’ of HIV to sex workers and then into the general population. Even if the prevalence of HIV among the general population remains low, given the size of the population, the absolute numbers of people living with HIV (PLHIV) could be very high. It is unlikely that the existing healthcare system would be able to cope with a large number of HIV-positive individuals, especially because what services exist are located in the main urban areas, although PLHIV are known to be distributed across the whole country and frequently in rural areas (National AIDS and STD Programme. Estimating numbers of those most affected by HIV/AIDS in different locations to support efficient service delivery, capacity building and community mobilization. 2009 [unpublished]).

**Table.** HIV/AIDS: the Bangladesh Scenario, 2009

Overall HIV prevalence among population groups most vulnerable to HIV infection	<1%
Reported number of HIV cases	1,745
Number of new HIV infections	250
Number of new AIDS cases	143
Estimated number of HIV infections	7,500

**Fig.**
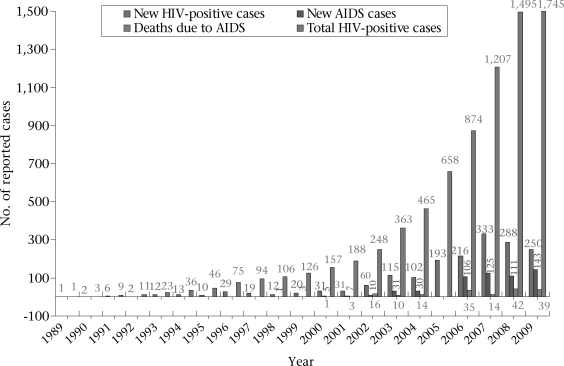
Number of reported HIV and AIDS cases, 1989–2009

Source: National AIDS and STD Programme, 2009

One of the five priority areas for the NSP is the provision of services and care for PLHIV, and yet for most PLHIV, the only accessible healthcare services are provided by non-governmental organizations (NGOs). NGOs provide a limited range of services, including voluntary counselling and testing, counselling, treatment for sexually transmitted infections, and outpatient-based management of medical problems, including opportunistic infections (National AIDS and STD Programme, 2009). The provision of antiretroviral drugs (ARVs) is patchy and depends on the NGO's funding and support as there is still no national provision of ARVs. ARVs are manufactured by two companies in Bangladesh but they provide only limited first-line treatment, and no paediatric formulations are available. CD4 counts and more complicated diagnostic tests are available only in Dhaka, and even in Dhaka, basic diagnostic tests for common opportunistic infections are not available (Matin N *et al.* Personal communication, 2010). The situation regarding hospitalization and care of acutely-ill patients is even worse. The Government of Bangladesh has a dedicated centre for treating PLHIV but this single ward is dependent on a single doctor who obviously cannot be available 24 hours a day. PLHIV frequently report that they do not disclose their status to healthcare providers for fear of stigma and of being denied care which further complicates the situation, but perhaps the greatest barrier to providing inpatient care to PLHIV is the lack of trained clinicians—doctors, nurses, counsellors, and others—and the lack of a team-based approach to the care of individuals in a holistic manner. As the case report by Pervez *et al.* in this issue of JHPN demonstrates, even well-meaning clinicians who are not used to treating PLHIV are likely to make understandable errors in the management of such individuals (3). It is against this background that the contribution of Matin and colleagues in describing the clinical epidemiology of severe disease among PLHIV in Bangladesh should be supported (Matin N *et al.* Personal communication, 2010).

While the national response of the Government of Bangladesh to the issue of HIV and AIDS, and of the donors which support it, has necessarily focused on community-based interventions, the time is fast approaching when large numbers of PLHIV will require hospital-based services. Unless the lack of antiretrovirals, diagnostics, and clinical capacity (in its broadest sense) is addressed quickly, a disaster looms.
